# Scaling Up Kangaroo Mother Care Through a Facility Delivery Model in Rural Districts of Pakistan: Protocol for a Mixed Methods Study

**DOI:** 10.2196/56142

**Published:** 2025-01-29

**Authors:** Shah Muhammad, Asif Soomro, Samia Ahmed Khan, Hina Najmi, Zahid Memon, Shabina Ariff, Sajid Soofi, Zufiqar Ahmed Bhutta

**Affiliations:** 1 Centre of Excellence in Women and Child Health Aga Khan University Karachi Pakistan; 2 Aga Khan University Hospital Institute of Global Health and Development Karachi Pakistan; 3 Aga Khan University Community Health Sciences Department Karachi Pakistan; 4 Hospital for Sick Children Toronto, ON Canada

**Keywords:** kangaroo mother care, scale up intervention, health facility, community, preterm infants

## Abstract

**Background:**

The neonatal mortality rate in Pakistan is the third highest in Asia, with 8.6 million preterm babies. These newborns require warmth, nutrition, and infection protection, typically provided by incubators. However, the high maintenance and repair costs of incubators pose a barrier to accessibility for many premature and low birth weight neonates in low- and middle-income countries. This study aims to implement a context-specific kangaroo mother care (KMC) model in Sanghar within secondary health care facilities and catchment communities.

**Objective:**

This study aims to achieve at least 80% KMC coverage for premature and low birth weight neonates.

**Methods:**

This research uses a mixed methods design grounded in implementation science principles, with the goal of developing adaptive strategies tailored to district and facility managers, as well as health care workers, leveraging previous evidence on the benefits of KMC. The research is conducted in the district of Sanghar, Sindh with an emphasis on promoting KMC for infants weighing between 1200 and 2500 g in three facilities. It includes preimplementation data collection, training of health care providers and lady health workers, and intervention involving mother-baby skin-to-skin contact, breastfeeding initiation, and postdischarge follow-ups. Ethical considerations and data management are prioritized, to improve KMC coverage and neonatal health outcomes.

**Results:**

This research will be implemented over a period of 18 months. The primary objective of this research is to achieve an 80% improvement in KMC coverage, with the secondary objective to promote optimal breastfeeding practices among postpartum mothers. Key indicators include the proportion of eligible infants enrolled in KMC, the percentage of mother-baby pairs receiving skin-to-skin care postdischarge, and the duration of KMC during the neonatal period. Additionally, the study will assess exclusive breastfeeding rates, neonatal weight gain, and neonatal deaths within the cohort. The data management team will evaluate the effectiveness of the model in achieving the targeted KMC coverage.

**Conclusions:**

The integration of KMC into the health care system will provide valuable insights for policy makers regarding effective implementation and scaling strategies. The study’s findings will highlight facilitators and barriers to KMC adoption, benefiting regions across Pakistan and globally. Additionally, these findings will offer valuable insights for the development of future newborn care programs.

**International Registered Report Identifier (IRRID):**

DERR1-10.2196/56142

## Introduction

Neonatal mortality continues to be a significant challenge for public health systems in low- and middle-income countries (LMICs), despite the global reduction in newborn deaths from 5 million in 1990 to 2.4 million in 2019. The majority of neonatal deaths occurred in India, Nigeria, and Pakistan [1]. Of these, 75% occurred during the first week of life, due to factors like preterm birth, intrapartum-related complications, low birth weight (LBW), birth asphyxia or lack of breathing at birth, infections, and congenital disabilities [2]. The leading cause of neonatal death is the preterm complication, defined as the live birth of a newborn before 37 weeks of gestation. An estimated 15 million babies are born prematurely every year, and approximately 1 million children die each year due to preterm birth complications. Additionally, many survivors face lifelong disabilities, including learning disabilities and visual and hearing impairments [3].

In Asia, Pakistan ranks third in neonatal mortality, with 8.6 million preterm babies. Premature and LBW neonates require warmth, adequate nutrition, and protection from infection [4]. Typically, these newborns are cared for using incubators, which are ventilated, sterile, moist, and oxygenated devices that provide life support by maintaining body temperature and ensuring a safe environment [5]. However, the maintenance and repair of incubators have significant financial implications, making this care modality challenging and inaccessible for most premature and LBW neonates in low- and middle-income countries, including Pakistan.

In Pakistan, 32% of neonates are born with LBW, representing one-third of all births in the country. In November 2015, the World Health Organization (WHO) identified kangaroo mother care (KMC) as the primary method of managing LBW and premature births [6]. The WHO has defined KMC as a practice of early, continuous, and prolonged skin-to-skin contact between mother and babies, in addition to exclusive breastfeeding or breastfeeding, early discharge after the onset of KMC in the hospital with continuation at home, and adequate support and follow-up for mothers at home [7].

The KMC strategy comprises three main components: thermal care by continuous skin-to-skin contact, support for exclusive breastfeeding or provision of other appropriate nutrition, and early detection and response to any complications that may arise. A systematic review has indicated that KMC is an effective and safe alternative to conventional neonatal care for LBW infants, especially in resource-limited settings [8]. For clinically stable LBW infants <2500 g, implementation of KMC has shown potential to reduce mortality, and if widely applied, could reduce premature deaths [9,10]. Globally, KMC has been recognized as an integral component of the newborn health initiative following the Every Newborn Action Plan [11]. WHO and the United Nations Children’s Fund have advocated for facility-based KMC as a routine method of care for clinically stable LBW newborns [12]. However, recent data from the Pakistan Demographic and Health Survey 2017-2018 revealed that only 6.3% and 6.5% of skilled birth attendants (SBAs) in rural Sindh and Balochistan, respectively, conducted newborn care practices that involved placing the newborn in skin-to-skin contact with the mother during childbirth [13].

In a recent study conducted by the Saving Newborn Lives program and Maternal and Child Health Integration Program, a situational analysis of 5 Asian countries, including Pakistan, was carried out to evaluate facility-based KMC [14]. Findings from the Maternal and Child Health Integration Program project implementation in Pakistan suggested that ensuring facility readiness, improved capacity of service providers at facility and community, and focused community mobilization effectively help program planners implement KMC at both levels of care [9,10]. India, on the one hand, has demonstrated substantial improvement in newborn and infant survival using a community-based KMC implementation and interpreted that community-initiated KMC substantially improves newborn baby and infant survival [7]. In Pakistan, researchers in newborn health have recently highlighted KMC as a top preterm intervention as part of the Every Newborn Action Plan [9,11]. Despite KMC being recognized as a cost-effective intervention, its adoption in Pakistan, both at the community and facility level, has been minimal.

The primary objective of this scaleup project is to introduce a context-specific KMC model in Sanghar focusing on secondary health care facilities and catchment communities, with the aim of attaining a minimum of 80% KMC coverage for premature and LBW neonates. Following the implementation of the KMC model in the targeted facilities and communities, the study also aims to assess the impact of this low-cost intervention on reducing neonatal morbidity and mortality among premature and LBW newborns within the rural health system of Sindh, Pakistan. This project will involve and encourage the adoption of the low-cost KMC model by public health facility leadership and community. Additionally, an implementation model tailored to the local context will be developed and assessed to achieve enhanced coverage. The findings of this study will hold significant implications for the government and other stakeholders in implementing and scaling up KMC at the district, provincial, or national level.

## Methods

### Study Design

A mixed methods design will be used with the principles of implementation science to develop an adaptive strategy to help district managers, facility managers, and health workers both at the facility and the community to identify ways to improve implementation. Considering the evidence from prior studies in Sindh and Pakistan proven to reduce the risk of hypothermia, improve the rate and duration of breastfeeding, improve early initiation of breastfeeding practices, improve mother-infant attachment and bonding, and reduce parental distress related to their infant’s well-being due to constant attachment [15-18], we aim to scale up the current practices for reaching over 80% coverage of KMC from the baseline. In the preimplementation phase of formative research, we will gather data on components of the health system and the organization of services, knowledge and skills of health care providers, and the community perceptions, acceptance, and challenges. Findings will be used to inform the development of an initial implementation model, addressing facility and community challenges, improving provider’s skills, and community acceptance, and improving service delivery to strengthen the health system services. This will be followed by regular assessments at different levels of KMC coverage.

We will implement the research in three facilities in District Sanghar. Our intervention promotes KMC for babies born weighing >1200 and <2500 g, both within the facility and its catchment area.

### Setting

The study will be conducted in Sanghar, which is in central Sindh; the total population of the district is 47.9 million, with 71% of the population residing in rural areas and 29% residing in urban areas. The district has a strong primary care setup with 60 basic health units and 6 rural health centers. Sanghar has 6 Mother and Child Health Centers and 2 maternity homes. The district is comprised of 38.3% public health facilities and 32.4% private health facilities, which translates into 70.7% institutional deliveries. Regarding assistance during delivery, District Sanghar has 72.2% of deliveries with SBAs compared to 82.7% of deliveries with SBAs in Sindh. The district reports poor maternal and child health indicators and a high neonatal mortality rate at 33 per 1000 live births, followed by infant and under-5 mortality rates at 47 and 56 per 1000 live births, respectively.

The intervention period for the study will be 18 months (from January 2022 to June 2023). Three public health facilities will be selected to adopt the KMC model. The selection of these facilities will be based on the availability of Maternal, Newborn, and Child Health services, as well as logistical considerations, such as availability of Basic Emergency Obstetric and Neonatal Care services, immediate referral capabilities, availability of human resources, and the monthly turnover of women seeking outpatient care for maternal health services, including the average number of deliveries (approximately 80-120) during the finalization of study sites.

### Study Population

#### Inclusion Exclusion Criteria

All stable newborns provided relevant consent of birth weight >1200 and <2500 g delivered at a targeted health facility or home in the catchment area of the correspondent health facility within District Sanghar will be considered for study. Newborns who are sick according to predefined criteria (ie, do not tolerate oral feeds, severe respiratory distress including respiratory rate of <20 breaths per minute, grunting, central cyanosis, very severe chest in-drawing, convulsions, unconsciousness, and severe hypothermia of <32 °C) will not be included. They will first be stabilized and referred to advanced care facilities. Newborns delivered outside the catchment area and those whose families did not provide consent will not be included.

#### Sample Size

We will include all newborns delivered in the selected facilities according to the inclusion and exclusion criteria over the 18-month implementation period.

### Intervention and Study Implementation Strategy

**Figure 1:**
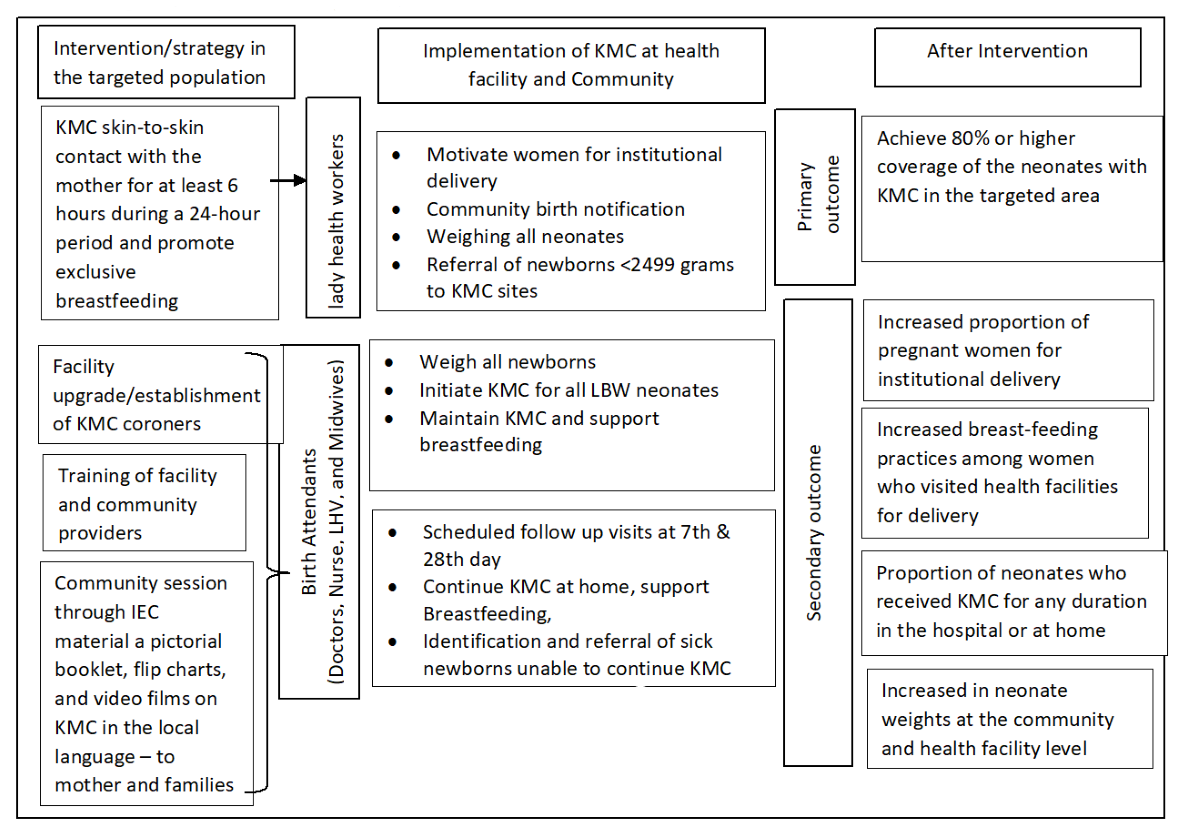
KMC implementation model. IEC: information, education, and communication; KMC: kangaroo mother care; LBW: low birth weight; LHV: lady health visitor.

The study will implement the KMC model in District Sanghar which involves skin-to-skin contact between the mother and the infant for a minimum of 6-8 hours within a 24-hour period, along with exclusive breastfeeding. Implementing the KMC delivery model (Figure 1) will take place in 3 stages: prefacility, facility, and postfacility. At each of these stages, various activities will be conducted, which include, enhancing the capacity of both the facility and community health care providers, establishing effective referrals from the community to health care facilities for institutional deliveries and KMC interventions, providing education on KMC to mothers, families, and the community to improve newborn health outcomes, and reinforcing follow-up procedures for both the community and health care facility.

All stable enrolled newborns (with relevant consent obtained) will be placed with skin-to-skin contact and breastfeeding will be initiated within the first hour of life. Mothers will be given tutorials on administering KMC by a trained physician or nurse or lady health visitor at the facility within 6 hours after delivery. At discharge, mothers and family members will be provided with counseling and encouraged to continue KMC practice at home.

### Formative Research Phase

We will use our (Umeed-e-Nau project) [10] project baseline data to assess KMC in the community, to review the current health care system, infrastructure, and resources available in the district. The available information will also help assess the existing newborn care practices, health care staff capacity, and community awareness about KMC. For successful KMC implementation, the study will identify key stakeholders involved in newborn care, including health care providers, administrators, policy makers, community leaders, and parents, and engage them in the planning process, ensuring their perspectives are considered and addressing any potential barriers to successful implementation. As part of our research planning, we will conduct interviews (focused group discussion and in-depth interviews with them to gain insights into the local context and factors influencing newborn care practices). In addition, we will also conduct observations to understand health system barriers, bottlenecks, community beliefs, knowledge, and attitudes toward KMC and further identify any cultural or social barriers that may need to be addressed. For this assessment, we will use the COM-B (Capability, Opportunity, Motivation, and Behavior) [18] to understand better the factors influencing behavior change within the community.

We will adopt a multipronged approach that involves active participation from both mothers and health care staff. This approach will address the various components required for behavior change: capability (knowledge or skill needed to perform a behavior), opportunity (a social environment that enables a behavior), and motivation (effective counseling and follow-ups to encourage and activate behavior change). Based on the findings from the formative phase, we will develop a plan for scaling up KMC across the targeted health facilities within District Sanghar.

### Intervention Phase

#### Prefacility Level

The prefacility-level activities will include training of health care providers, and promoting facility birth by involving lady health workers (LHWs) to refer pregnant women for institutional delivery. This has already been implemented by LHWs, who provide immediate referrals for LBW babies delivered at home or in other facilities through KMC counseling.

Counseling during antenatal care visits by LHWs will help increase institutional deliveries and facilitate intrafacility referrals of LBWs to KMC sites. Each month, LHWs will prepare a list of pregnant women as part of their routine activities, submitting this list with their monthly reports to their respective lady health supervisors. Data collectors will validate this information during their monthly visits to the LHWs.

Pregnant women in the community will be encouraged to visit health facilities for antenatal care and institutional delivery through the LHWs, as this is part of their mandate. This antenatal visit and counseling will also include information about KMC. This activity aims to identify the total number of pregnant women who can be referred to the facility for delivery and KMC if their neonates meet the inclusion criteria of weighing >1200 and <2500 g.

All neonates will be screened at birth, and those with a birth weight of >1200 and <2500 g will be referred to the KMC functional site in their respective tehsils or districts. The KMC intervention will be initiated with all LBW babies meeting the inclusion criteria who are delivered within the study catchment area, either at the KMC site or by referral to the KMC sites.

#### Training of Health Care Staff

**Figure 2:**
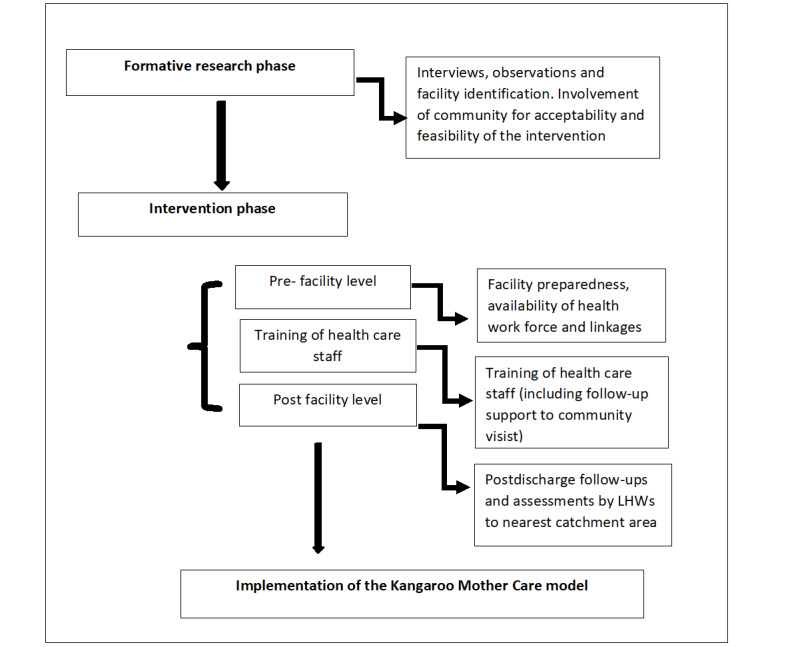
Flow diagram for study implementation. LHW: lady health worker.

KMC experts from Aga Khan University (AKU) will serve as master trainers for the training, based on educational material on KMC in the local language for mothers, families, health facility providers (physicians, nursing staff, and lady health visitor staff), LHWs, and study staff at the district level. The training routine will consist of a 1-day classroom and 2-day real-life field scenario for facility providers. Similarly, the LHWs of the selected facility catchment area will also be trained for institutional delivery referral, KMC case notification, and follow-ups. They will be trained to deliver information, education, and support for the KMC practices at the household level in a 2-day training workshop by respective health facility providers. At discharge, mothers and family members will be counseled so that KMC can be continued at home. Facility staff will notify the data collector of discharges and schedule follow-up visits on the 7th and 28th day (Figure 2).

#### Postfacility Level

Community-based KMC will be administered at home by LHWs exclusively to enrolled neonates discharged from facilities and those born at home within the catchment population of a designated KMC facility. Postdischarge, follow-up will be conducted by respective LHWs. They will visit the home on the 7th and 28th days to assess the implementation of the postfacility KMC protocol. The follow-up team will continue to monitor the newborn at each visit, using a checklist to assess KMC practices, including skin-to-skin contact duration (hours per day) and breastfeeding status, and recording the newborn’s weight. Additionally, the team will identify and refer sick newborns to the facility and assist with the referral process. This approach, aligned with the health systems strengthening component (continuum of care), aims to create a linkage between facility and community, encouraging the continuation of KMC at home.

#### Implementation of KMC (The Delivery Model)

**Figure 3:**
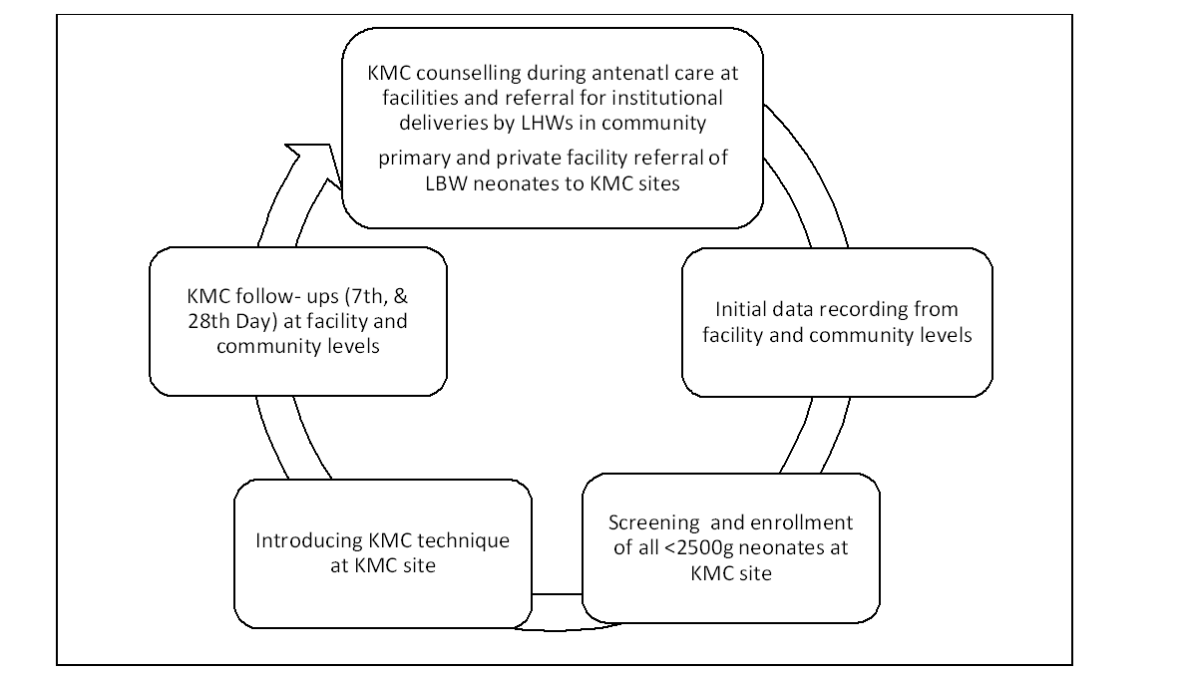
Continuous scale-up model of KMC, Umeed-e-Nau, at the facility level. ANC: antenatal care; KMC: kangaroo mother care; LBW: low birth weight; LHW: lady health worker.

The most effective KMC delivery strategy will be determined based on program findings. Data will be gathered from all established KMC sites to assess coverage and adherence to KMC guidelines, including the utilization of communication and training materials and the effectiveness of implementation tools. The results on progress and compliance with process indicators will be shared at the District Health Program Management Team meeting. This meeting will convene key stakeholders, such as health facility managers, LHW program focal persons responsible for managing outreach components, and other individuals involved in the design, implementation, and rollout of the KMC delivery model. Continuous collaboration with these provincial-level stakeholders will enable the integration of best practices and adjustments at the facility level to promote effective KMC and its sustained benefits for vulnerable populations (Figure 3).

### Data Collection Teams

The KMC intervention will be carried out by health workers, including facility staff and LHWs, employed by the district or provincial government at both health facility and community levels. The role of the AKU research team in implementation will primarily be supportive, initially focusing on the formulation of the study design and ongoing process assessment for monitoring and evaluating the intervention’s outcomes in the target population. The AKU team will also facilitate data collection by conducting interviews with various stakeholders, such as health care providers, administrators, policy makers, community leaders, and parents, to evaluate the feasibility and acceptability of the intervention. Additionally, the team will provide quality assurance and guidance to health facility staff and community-based health care providers to ensure the effective execution of the KMC intervention within a supportive environment.

### Data Management

The collected dataset will be linked to participant identification numbers, separate for mothers and newborns. The cleaned dataset will be presented in monthly review meetings with the principal investigator for review and feedback. Data quality assurance methods would include digital real-time checks for missing values and inconsistency, random spot checks, and data validation coupled with independent monthly audits. The collected data will be stored in a password-protected file and only the data manager from the data management unit will be allowed to access the data and will share the analysis elsewhere needed.

### Ethical Considerations

The study was approved by the ethical review committee of the AKU, Karachi-Pakistan (2021-6444-19970). Before enrollment into the study, a verbal explanation of the research will be provided, and written consent will be obtained from the mother or an adult family member. In cases where the participant cannot read the informed consent form, a research team member will read it out to them before recruitment. The participants will be fully informed that they have the right to withdraw from the study at any time, without any negative consequences or compensation, and their decision to withdraw will not impact their access to any services provided by the facility. The study will be scaled up within the existing health system so monetary compensation will be provided. However, all the LBW neonates enrolled in the study will be provided with standardized KMC kit which includes, a shirt for mother and baby suites 3 zero size, diapers, woolen cap, socks, soap bar for neonates.

We will also maintain the participant’s confidentiality during our research, all identified information of the study participants will be kept confidential and can only be accessed by the principal investigators or supervisors. The findings will be conveyed to the communities through the health system’s channels, as well as district, provincial, and national health authorities will be informed through project dissemination and publication in peer-reviewed journals.

## Results

### Expected Results

Through the implementation of this research, our results include achieving an 80% improvement in KMC coverage and promoting optimal breastfeeding practices among postpartum mothers. The data collection of our research is completed and so far, 12499 live births have been reported and we enrolled 3046 LBWs for KMC and we successfully followed up all registered neonates. We are currently cleaning and refining our data sets for analysis and the actual results of our research will be available in the original research article. The key indicators to be measured include the proportion of eligible infants enrolled in KMC, the percentage receiving skin-to-skin care within specified timeframes post discharge, and the duration of KMC during the neonatal period. Additionally, the study will assess the proportion of eligible infants exclusively breastfed at various intervals, along with monitoring neonatal weight gain and tracking neonatal deaths within the enrolled cohort. The data management team will evaluate the model’s effectiveness in achieving the targeted KMC coverage among the entire study population, as outlined in the outcome measurement section.

### Evaluation

Effective KMC coverage is defined as the number of newborns receiving KMC divided by the total number of newborns eligible for KMC (>1200 and <2500 g) at the facility and communities during the evaluation period. To measure the denominator for effective KMC coverage, it is preferred that all facility births be identified during facility visits by reviewing birth registers from all facilities. In addition, the data collector will liaise with LHWs to obtain the number of home births and their birth weights. To calculate the numerator for effective KMC coverage, the data management team will quantify the number of newborns weighing >1200 and <2500 g who received KMC in the study population. All enrolled newborns will be followed by the data collector in the facility or at home to collect data at two points: the 7th and 28th day of initiation of KMC. At each time point, the team will record the data through the KMC checklist. We will disseminate the scaling-up findings of KMC across facilities in Sanghar districts with further dissemination of this scale-up at the provincial and national levels to encourage its implementation within other provinces.

## Discussion

### Increasing Coverage for Premature and LBW Neonates

This will be the first implementation research project to develop and evaluate scale-up models for achieving high population coverage with KMC in Pakistan. Implementation of a context-specific KMC model in Sanghar within secondary health care facilities and surrounding communities will help to achieve at least 80% KMC coverage for premature and LBW neonates. The model in the targeted facilities and communities will also assess the impact of this low-cost intervention on reducing neonatal morbidity and mortality among premature and LBW newborns within the rural health system of Sindh, Pakistan.

This project is a collaborative initiative between AKU and the provincial government, aiming to evaluate the effectiveness of scaling up KMC coverage. Preliminary data indicates that KMC is currently practiced for <5% of LBW infants in these settings, with no competing interventions promoting KMC at the study sites [6]. There is existing literature on the barriers and facilitators of KMC within health facilities [19-21]. This project presents a timely opportunity to integrate KMC into the existing health care system in partnership with the government. The goal is to identify effective scale-up models for implementation in large, countrywide settings. While the study focuses on KMC coverage as a key outcome, we aim to develop a model that integrates KMC as an essential component of newborn care, rather than projecting it as a standalone vertical program.

The study will exclude community-based follow-ups to evaluate improvements in newborn weight for families that do not provide consent for such follow-ups. Further, the accuracy and reliability of outcome measurements, such as growth parameters and developmental milestones, were not used.

### Conclusions

The outcomes of this implementation research will extend beyond the study area to benefit other regions within Pakistan and globally. The findings will inform strategies to enhance essential newborn care provision at health care facilities, seamlessly integrating KMC without the need for a separate vertical program. Insights gained from this research will not only illuminate the challenges associated with scaling up KMC but also offer effective solutions to address them. Furthermore, these experiences hold promise for shaping the design and implementation of future infant and childcare programs, facilitating their swift adoption by both health systems and communities. The findings of this study will provide robust evidence to inform the development of policies and programs aimed at preventing neonatal mortality and improving maternal and child health and growth outcomes in resource-limited settings.

## Abbreviations

AKU: Aga Khan University
COM-B: Capability, Opportunity, Motivation, and Behavior
KMC: kangaroo mother care
LBW: low birth weight
LHW: lady health worker
SBA: skilled birth attendant
WHO: World Health Organization
